# A Biocompatible, Highly Sensitive, and Non-Enzymatic Glucose Electrochemical Sensor Based on a Copper-Cysteamine (Cu-Cy)/Chitosan-Modified Electrode

**DOI:** 10.3390/nano14171430

**Published:** 2024-08-31

**Authors:** Huan Chen, Tingting Gu, Longyang Lv, Xing Chen, Qifeng Lu, Amer Kotb, Wei Chen

**Affiliations:** 1 School of Chemical Engineering, University of Science and Technology Liaoning, Anshan 114051, China; ch1120@stu.ustl.edu.cn (H.C.); lvlongyang@stu.ustl.edu.cn (L.L.); chenxing@stu.ustl.edu.cn (X.C.); 2 School of Chips, XJTLU Entrepreneur College (Taicang), Xi’an Jiaotong-Liverpool University, Taicang, Suzhou 215400, China; qifeng.lu@xjtlu.edu.cn (Q.L.); amer.kotb@xjtlu.edu.cn (A.K.)

**Keywords:** copper-cysteamine (Cu-Cy), electrochemical sensor, glucose, enzyme-free

## Abstract

A biocompatible, highly sensitive, and enzyme-free glucose electrochemical sensor was developed based on a copper-cysteamine (Cu-Cy)-modified electrode. The catalytically active biocompatible material Cu-Cy was immobilized on the electrode surface by the natural polymer chitosan (CTS). The electrochemical characterization and glucose response of the Cu-Cy/CTS/glassy carbon electrode (GCE) were investigated by electrochemical impedance spectroscopy (EIS), cyclic voltammetry (CV), and constant potential amperometry. The significant electrocatalytic activity of Cu-Cy to the oxidation of glucose in an alkaline environment was revealed. Several crucial parameters, including the number of scanning cycles for electrode activation, applied potential, and the contents of Cu-Cy and chitosan, were investigated to understand their impact on the sensor’s response. The proposed sensing platform exhibited linear ranges of 2.7 μM to 1.3 mM and 1.3 mM to 7.7 mM for glucose detection, coupled with high sensitivity (588.28 and 124.42 μA·mM^−1^·cm^−2^), and commendable selectivity and stability. Moreover, a Cu-Cy/CTS-modified screen-printed electrode (SPE) was further developed for portable direct detection of glucose in real samples.

## 1. Introduction

Persistent high blood sugar can lead to extensive damage to the body’s blood vessels, impacting vital organs such as the heart, eyes, kidneys, and nervous system, which may result in serious health complications [1,2]. Diabetes poses a significant threat to global health due to its potential to cause severe complications, including cardiovascular diseases, renal dysfunction, and vision loss [3]. The development of highly sensitive and highly selective glucose assays is important in the clinical diagnostics [4], pharmaceutical [5], and food industries [6]. Nowadays, some methods are being used for glucose detection, such as liquid chromatography, spectrophotometry, and colorimetric analysis [7,8,9]. Despite their drawbacks, such as the intricate procedures involved in sample preparation and pretreatment, the relatively slower pace of analysis, and the challenges associated with conducting on-site detection, these methods have their limitations. On the other hand, electrochemical techniques have been gaining considerable attention in the research and application fields due to their numerous benefits. These include their cost-efficiency, the ability to provide quick results, user friendliness, heightened sensitivity, and the capability for on-site analysis [10].

Electrochemical methods for glucose detection can be classified into two categories depending on the presence of glucose oxidase (GO_x_) on the electrode surface: enzyme-based and enzyme-free glucose electrochemical sensors [11]. Though enzyme-based sensors benefit from the high selectivity of GO_x_ for glucose, the inherent limitations such as high cost, lesser environmental stability, and storage difficulties hinder their potential applications [12]. Consequently, research has been directed towards non-enzymatic glucose sensors as a viable alternative. The main principle of these electrochemical sensors is based on the direct electrochemical oxidation of glucose at the electrode surface [13]. The lack of enzymes in these sensors not only lowers the cost and enhances stability but also simplifies the sensor preparation process. A variety of electrode materials have been identified as promising options for catalytic roles in non-enzymatic glucose sensors, as noted in [14]. Electrocatalytic glucose-modified electrode materials span across several chemical elements, including transition metal nanomaterials like Ni, Co, and Cu [15,16], noble metal nanomaterials such as Au, Ag, and Pt [17,18], and carbon-based composite electrode materials including carbon nanotubes and graphene [19,20], among others. Among these, Cu-based composites are often utilized in the field of electrochemical glucose sensors due to their robust electrocatalytic activity. Although noble metals like Pt and Au are superior to Cu-based materials in aspects such as electrical conductivity and catalytic activity, Cu is favored for applications due to its lower cost [21]. With the ongoing advancements in fabrication techniques, Cu-based glucose electrochemical sensors developed from a range of materials are becoming a burgeoning area of research. In a study conducted by T. Sridara and colleagues [22], they developed an enzyme-free glucose sensor utilizing a nanocomposite of carbon nanodots and copper oxide. This sensor demonstrated a high sensitivity for glucose detection, with two distinct linear response ranges: 0.5 to 2 mM and 2 to 5 mM, and respective sensitivities of 110 and 63.3 µA mM^−1^cm^−2^. S. Ayaz and colleagues [23] utilized Cu nanoparticle-modified graphite pen electrodes for the detection of glucose through an enzyme-free, flow-injection amperometric technique. The technique provided a detection range from 0.10 to 400 µM, a detection limit as low as 0.04 µM, and exhibited a sensitivity of 830 µA mM^−1^ cm^−2^. Currently, Cu-based materials for enzyme-free detection of glucose are difficult to achieve biocompatibility, which to a certain extent limits the application of such sensors in future detection.

Cysteamine, acknowledged as the most uncomplicated stable aminothiol, originates from the degradation of the amino acid cysteine and finds widespread application in medical treatments, as documented in [24]. Its strong metal-binding capacity initiates a cascade of chemical reactions with a variety of transition metals, thereby fostering the creation of innovative nanomaterials, including copper-cysteamine (Cu-Cy) [25,26]. Cu-Cy, as a new photosensitive nanomaterial, has shown excellent performance in the optical field and cancer treatment, which can be simply obtained through a green chemistry process [27]. On the other hand, CTS is a natural cationic polysaccharide that is rich in -OH and -NH_2_ groups. Given its distinctive characteristics, including its high biocompatibility, ease of forming films, hydrogels, or sponges, and notable toughness [28], CTS has become a subject of extensive research for a range of applications. This includes the development of electrochemical and biosensors, with a significant concentration on use in the pharmaceutical industry and clinical diagnostics [29]. It has been observed that integrating CTS with a variety of nanomaterials substantially improves the sensitivity and detection efficiency of electrochemical sensors [30,31]. In this study, a biocompatible, highly sensitive, simple, and enzyme-free glucose electrochemical sensor was proposed based on a Cu-Cy/CTS-modified electrode. The catalytically active biocompatible material Cu-Cy was firstly immobilized on the electrode surface by the natural polymer CTS. The electrochemical catalytic activity of the Cu-Cy-modified electrode on glucose and various characteristics of the sensor were investigated. A Cu-Cy/CTS-modified SPE was further developed for portable direct detection of glucose in real samples. The Cu-Cy/CTS-modified electrodes present a promising avenue for the advancement of future electrochemical sensors and biosensors, characterized by their biocompatibility, affordability, sensitivity, selectivity, ease of use, and rapid response to a variety of analytes.

## 2. Experimental Details

### 2.1. Chemicals

The Cu-Cy used in this study was synthesized following the method detailed in reference [26]. The chemicals used, including glucose, CTS, galactose, uric acid, and ascorbic acid, were sourced from Beijing Solarbio Science & Technology Co., Ltd. (Beijing, China). Lactic acid was procured from Tokyo Chemical Industry (Tokyo, Japan). All the solutions utilized in this investigation were prepared using Millipore water (Burlington, MA, USA).

### 2.2. Instruments

Electrochemical measurements were carried out on a CHI660E electrochemical workstation (Chenhua Apparatus Co., Shanghai, China). The morphology of the samples was characterized by FESEM (Sigma 500, Carl Zeiss AG, Jena, Germany).

### 2.3. Preparation of Cu-Cy-Modified Electrodes

Prior to modification, GC electrodes (bought from CH Instruments, Inc., Austin, TX, USA, diameter 3 mm) were polished with deionized water, 0.5 and 0.05 μm Al_2_O_3_, respectively. Then, 30 μL of Cu-Cy solution (1 mg/mL), which was sonicated for 900 s before use, and 5 μL of CTS solution (2 mg/mL) were dropped onto the GCE surface by a micropipette. The electrode was air-dried at room temperature (20–22 °C) under a 1000 mL capacity beaker for 22 to 24 h [32]. Before electrochemical measurements, the Cu-Cy/CTS/GCE was activated in a 0.1 M NaOH solution (30 mL) by cyclic voltammetry, scanning for 50 cycles at a potential range from −1.0 V to +1.0 V with a scan rate of 50 mV/s and without the need for a stabilization time before starting the scans. For blank contrast experiments, the modified electrode was prepared using the corresponding solution, adhering to the same electrode preparation method. The modified electrodes were stored in the refrigerator at 4 °C. The modification process of the screen-printed carbon electrode (Weihai Poten Technology Co., Ltd., Weihai, China, diameter 4 mm) was the same as that of GCE, except that polishing was not required before the modification.

### 2.4. Electrochemical Measurements

All experiments were carried out in a standard three-electrode in 30 mL of a 0.1 M NaOH solution. A platinum wire electrode acted as the counter electrode, while an Ag/AgCl electrode served as the reference electrode. Cu-Cy/CTS/GCE served as the working electrode. Both constant potential amperometry (I-T) and cyclic voltammetry (CV) were applied to measure glucose oxidation at the CHI 660 E electrochemical workstation. The constant potential amperometry was then used to detect different concentrations of glucose. Under constant stirring, the current response of glucose on the electrode was observed and recorded with the continued addition of glucose. EIS measurements were performed in a 0.1 M KCl solution containing 10 mM [Fe(CN)_6_]^3−/4−^ with a frequency range of 0.01–100,000 Hz. The detection potential for EIS was determined by measuring the open-circuit potential of each modified electrode before each test.

### 2.5. Analysis of Real Samples

Serum samples obtained from Shanghai Titan Scientific Co., Ltd. (Shanghai, China), did not require preprocessing and were stored at −20 °C. According to the literature [33,34], the standard addition method was used in subsequent measurements. The spiked recoveries were obtained using the formula (*C*2 − *C*1)/*C*3 × 100% [35], where *C*1 was the glucose concentration in the serum detected by the sensor, *C*2 was the glucose concentration in the serum sample after the addition of glucose standard solution, as detected by the sensor, and *C*3 was the concentration of the glucose standard solution added above.

## 3. Results and Discussion

### 3.1. Characterization and Electrochemical Behaviors of Cu-Cy/CTS/GCE

**Figure 1:**
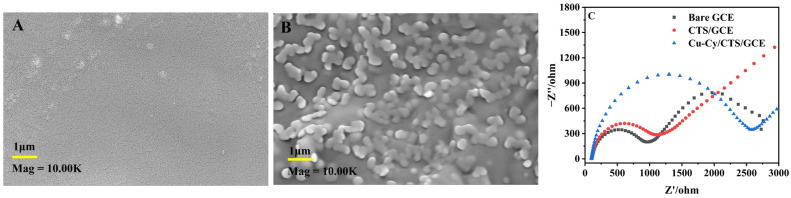
SEM images of CTS film (**A**) and Cu-Cy/CTS film (**B**), and (**C**) EIS of bare GCE, CTS/GCE, and Cu-Cy/CTS/GCE in a 0.1 M KCl solution containing 10 mM [Fe(CN)_6_]^3−/4−^.

The morphology of the electrode surface was characterized by scanning electron microscopy (SEM), as shown in Figure 1. The surface photo of the CTS film (Figure 1A) shows a uniform film appearance, indicative of chitosan’s good film-forming property [35]. In contrast, the Cu-Cy/CTS film (Figure 1B) displayed a film structure that evenly encapsulated small particles. A comparative analysis with the CTS film confirmed that these particles were Cu-Cy. The size of the observed Cu-Cy particles was approximately 300 nm, larger than sizes typically reported in the literature [36]. The observed inconsistency could be due to the Cu-Cy aggregating on the electrode’s surface during the film formation process. This aggregation, coupled with the surface adsorption effects with chitosan, likely contributes to the observed increase in particle size. The uniform and stable immobilization of Cu-Cy on the electrode surface by chitosan likely contributes to the enhanced subsequent electrochemical activity.

The impedance change of the surface-modified electrode was examined using an electrochemical working station, carried out at room temperature. As depicted in Figure 1C, the Nyquist plot presents the EIS of diverse electrodes immersed in a 0.1 M KCl solution containing 10 mM [Fe(CN)_6_]^3−/4−^, across a frequency range of 0.01 Hz–100,000 Hz. In the Nyquist plot, the semicircle’s radius is indicative of the resistance to electron transfer (R_ct_), serving as a measure of both the material’s electrical conductance and the kinetics of electron exchange [33]. The R_ct_ was calculated from the semicircle diameter, and the fitting about the semicircle was performed automatically by the CHI660E software. In this case, the R_ct_ of the bare electrode stood at 948 Ω, the R_ct_ of CTS/GCE was noted at 1127 Ω, and the R_ct_ of Cu-Cy/CTS/GCE reached 2582 Ω. The bare GCE show a lower R_ct_ compared to the CTS/GCE, which can be attributed to the inferior electrochemical activity of CTS. The R_ct_ observed for the Cu-Cy/CTS electrode was higher compared to that of the CTS/GCE, indicating successful assembly of Cu-Cy nanoparticles on the electrode surface. Cu-Cy inhibited the electron transfer from the redox probe, [Fe(CN)_6_]^3−/4−^, which was similar to the properties of semiconductor-like copper oxides (CuO, Cu_2_O), as reported [33]. Copper is an excellent conductor of electricity, but the conductivity of Cu-Cy materials may be influenced by factors such as the content of copper, the coordination state of cysteamine, and the microstructure of the composite material.

**Figure 2:**
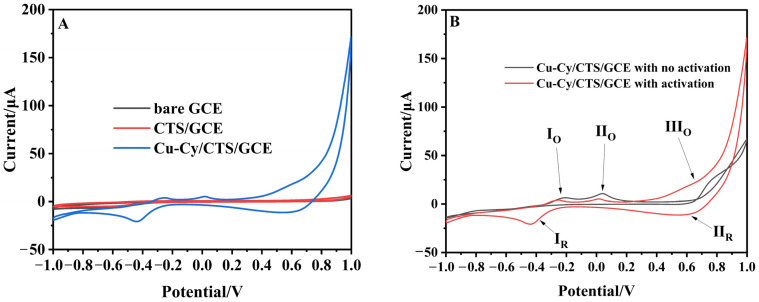
(**A**) CVs of bare GCE, CTS/GCE, and Cu-Cy/CTS/GCE in sodium hydroxide solution; (**B**) CV of Cu-Cy/CTS/GCE before and after activation in 0.1 M NaOH (scan rate: 50 mV/s).

The redox reaction occurring between the electrode surface and electrolyte can be qualitatively and quantitatively verified via cyclic voltammetry. Figure 2A presents the cyclic voltammetric curves of bare GCE, CTS/GCE, and Cu-Cy/CTS/GCE in sodium hydroxide solution. Under the experimental conditions, no distinct redox peaks were observed for bare GCE and CTS/GCE. For the Cu-Cy/CTS/GCE, shown in Figure 2B, the oxidation peaks I_O_ and II_O_, observed around −0.247 V and 0.017 V, respectively, along with the reduction peak IR, formed by the fusion of two reduction peaks, were evident around −0.441 V. Cu-Cy material mainly contained Cu(I) [25]. These peaks on the Cu-Cy/CTS/GCE may be due to the redox pairs Cu(0)/Cu(I) and Cu(I)/Cu(II). Furthermore, a redox pair observed close to 0.615 V (III_O_ and II_R_) could be attributed to the Cu(II)/Cu(III) redox process [37]. Figure 2B displayed a comparison of the CV of the Cu-Cy/CTS/GCE before and after activation. The Cu(II)/Cu(III) redox pair emerged as a result of electrochemical activation in a sodium hydroxide solution. The presence of an alkaline environment enabled the electrochemical conversion of Cu(I) in Cu-Cy to Cu(II) and further to Cu(III), a critical step for the efficient electrooxidation of glucose [37,38].

### 3.2. Electrochemical Response of Cu-Cy/CTS/GCE to Glucose

**Figure 3:**
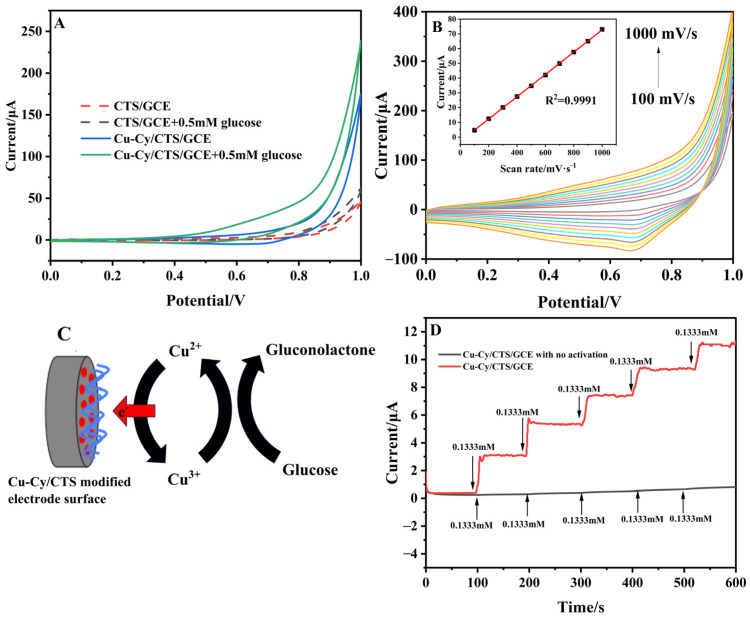
(**A**) CVs of Cu-Cy/CTS/GCE (solid lines) and CTS/GCE (dotted lines) in the absence and in the presence of 0.5 mM concentration of glucose in 0.1 M NaOH (scan rate: 50 mV/s); (**B**) CVs of t Cu-Cy/CTS/GCE at different scan rates from 100 to 1000 mV/s in 0.1 M NaOH containing 1 mM glucose. Inset shows the relationship of the oxidation peak current of glucose with the scan rate; (**C**) Schematic diagram of glucose oxidation mechanism involved at Cu-Cy/CTS/GCE; (**D**) Amperometric response of the Cu-Cy/CTS/GCE with successive addition of glucose into 0.1 M NaOH at 0.55 V.

Figure 3A illustrates the solid lines, which represent the CVs of the Cu-Cy/CTS/GCE with and without glucose. The addition of glucose resulted in a noticeable increase in the oxidation current of the Cu-Cy/CTS/GCE at 0.615 V, rising from 2.5 μA in the absence of glucose to 28.5 μA in the presence of 0.5 mM glucose. At the same time, the reduction peak current decreased, which was consistent with the characteristics of electrochemical catalysis. The potential oxidation mechanism of glucose at Cu-Cy/CTS/GCE can be explained in the following steps [39,40]. As shown in Figure 3C, Cu (III) on the surface of the electrode catalyzed the oxidation of glucose to gluconolactone in the solution while simultaneously being reduced to Cu (II). Subsequently, within the electrode layer, Cu (II) gave electrons to the electrode and reverted back to Cu (III), leading to a significant increase in the peak anodic current of CV on the electrode. As a contrast, the dotted lines shown in Figure 3A were the CVs of CTS/GCE with and without glucose. The CV of CTS/GCE show no obvious change of oxidation peak. Furthermore, the relationship between the oxidation peak current of the Cu-Cy/CTS/GCE in the presence of glucose and the scan rate was investigated. As shown in Figure 3B, the peak currents increased with the rising scan rate, exhibiting a linear relationship with the scan rates, as illustrated in the inset of Figure 3B. The linear regression equation was expressed as *Ip* = 0.07543*v* − 2.76 (R^2^ = 0.999). This result indicated that the electrocatalytic oxidation of glucose at the Cu-Cy/CTS/GCE was controlled by the electrochemical reaction occurring on the electrode surface, which was consistent with the findings in the absence of glucose, as depicted in Figure S1A. Since Cu-Cy was the only electroactive substance on the Cu-Cy/CTS/GCE, it was stably immobilized on the electrode surface. There was no need for Cu-Cy to diffuse to the electrode surface from the solution. Therefore, the limitation of the catalytic reaction comes from the electrochemical reaction of Cu-Cy on the electrode surface. Then, we further investigated the Cu-Cy/CTS/GCE’s ability to electrocatalyze glucose by amperomeric determination. As shown in Figure 3D, the amperometric response increased significantly with the increase in glucose concentration. As a comparison, it can be found that the modified electrode with no activation has little response to glucose. These findings suggested that the Cu-Cy/CTS/GCE electrode exhibits promising electrocatalytic performance for glucose detection, coupled with a rapid response time of less than 10 s.

**Figure 4:**
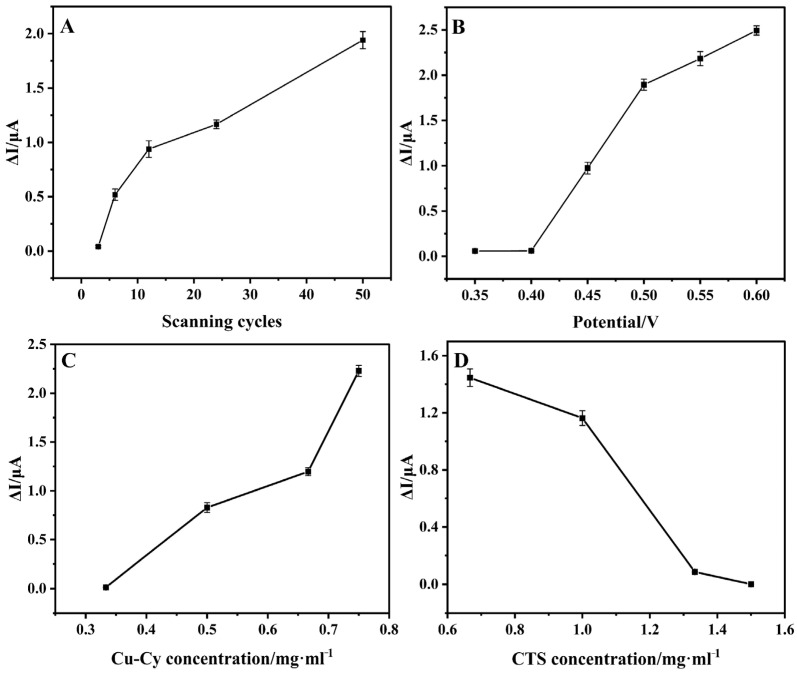
The current measurement of Cu-Cy/CTS/GCE in 0.1333 mM glucose (**A**) with the number of scanning cycles for electrode activation, (**B**) with varying detection potential, (**C**) with different concentrations of Cu-Cy, and (**D**) with different concentrations of CTS.

Various experimental factors, such as the number of scanning cycles for electrode activation, applied potential, and the content of Cu-Cy and CTS, were investigated to optimize the analytical properties of the Cu-Cy/CTS/GCE for glucose detection. All the experiments were conducted in glucose solution with a concentration of 0.1333 mM, and the concentration fell within the clinical relevance of the concentration range. As shown in Figure 4A and Figure S2A, the current response of the Cu-Cy/CTS/GCE to glucose amplified as the number of scanning cycles for electrode pre-activation increased from 3 to 50. As previously mentioned, the formation of Cu(III) was crucial for the electrochemical catalysis of glucose. CV curves of Cu-Cy/CTS/GCE activated for different numbers of cycles in the absence of glucose in Figure S1B show that increasing the number of activation cycles can greatly improve the redox current of the Cu(II)/Cu(III) couple. That is to say, the activation for the electrode increased the amount of Cu(III) and electrochemical activity of Cu-Cy/CTS/GCE, thereby enhancing the catalytic current for glucose. Figure 4B and Figure S2B show the effect of detection potential on the current response of the Cu-Cy/CTS/GCE in the range from 0.35 V to 0.6 V. The maximum electrocatalytic activity of Cu-Cy/CTS/GCE towards glucose oxidation was achieved at a detection potential of 0.6 V. However, using high applied potentials can potentially introduce background noises due to enhanced sensitivity to interferences, such as ascorbic acid and uric acid, typically found in human blood or serum [41,42]. Therefore, an optimal operating potential of 0.55 V was selected. Moreover, the influence of the Cu-Cy ratio in Cu-Cy/CTS on the current response was investigated, as displayed in Figure 4C,D and Figure S2C,D. The peak currents at the Cu-Cy/CTS/GCE increased as the concentration of Cu-Cy rose from 0.33 mg/mL to 0.75 mg/mL, which may be attributed to the increased active sites on the modified electrode. This was supported by the observation that the peak area of the Cu(II)/Cu(III) redox peaks increased with the concentration of Cu-Cy, as shown in Figure S1C. Conversely, as the concentration of CTS increased from 0.67 mg/mL to 1.5 mg/mL, the peak currents at the Cu-Cy/CTS/GCE decreased, which was due to the reduced electrode conductivity and the longer electron transfer distance from the solution to the electrode surface with a high CTS ratio.

### 3.3. The Performance of the Amperometirc Glucose Sensors

**Figure 5:**
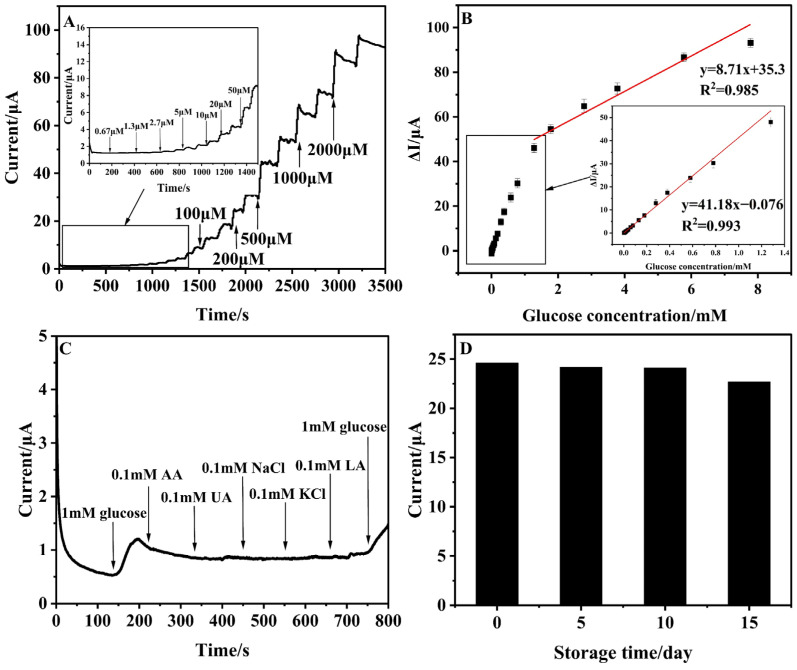
(**A**) Amperometric response of the Cu-Cy/CTS/GCE electrode upon the addition of various concentrations of glucose in 0.1 M NaOH at 0.55 V; (**B**) The corresponding calibration curve of the Cu-Cy/CTS/GCE; (**C**) Amperometric response of Cu-Cy/CTS/GCE for the successive addition of 1 mM of glucose and 0.1 mM of AA, UA, NaCl, KCl, lactic acid, and glucose in 0.1 M NaOH at 0.55 V. (**D**) Long-term stability studies of Cu-Cy/CTS/GCE in the determination of glucose (0.9 mM).

Amperometric measurements were utilized for glucose detection, employing a Cu-Cy/CTS/GCE as the working electrode at a constant oxidation potential of +0.55 V, which immobilized 0.75 mg/mL of Cu-Cy and 0.67 mg/mL of CTS with 50 scanning cycles of activation. The current increased with increasing glucose concentration, as shown in Figure 5A. In Figure 5B, the calibration curve outlined the relationship between the current response and glucose concentration. Here, the Cu-Cy/CTS/GCE demonstrated a linear response in two different glucose concentration ranges, 2.7 μM–1.3 mM (R^2^ = 0.993) and 1.3–7.7 mM (R^2^ = 0.985), with a current sensitivity of 588.28 μA mM^−1^cm^−2^ and 124.42 μA mM^−1^cm^−2^, respectively, and a LOD of 0.28 μM. The observation of two linear ranges in the calibration curve was attributed to the different oxidation states of glucose and its interaction with the sensing material [34,43]. Key parameters of the Cu-Cy/CTS/GCE were further compared with previous enzyme-free Cu-based glucose sensors, as shown in Table 1. Compared with other Cu-based non-enzymatic glucose sensors, the current work introduces a glucose sensor that exhibits superior sensitivity, despite its detection limit not surpassing that of other materials.

In order to validate the anti-interference capabilities of the electrodes, we performed selective measurements of 1 mM glucose in the presence of 0.1 mM physiological interfering compounds such as ascorbic acid (AA), uric acid (UA), lactic acid (LA), NaCl, and KCl in 0.1 M NaOH at 0.55 V. Typically, in human blood samples, the glucose concentration significantly exceeds that of other interfering substances [50]. As demonstrated in Figure 5C, the currents induced by physiological interferents (AA, UA, NaCl, KCl, and LA) were marginal when compared to the robust and pronounced response by glucose.

Reproducibility, repeatability, and stability were vital attributes for any electrochemical sensor [33]. In this experiment, reproducibility was the assessment of the error between immobilizing the same film for the same experiment with five Cu-Cy/CTS/GC electrodes, prepared independently. The relative standard error (RSD) of 1.4% for the detection of 0.9 mM glucose under the same condition was obtained, indicating that the sensor had good repeatability (Figure S3A). Furthermore, the sensor’s repeatability was commendable, as evidenced by an RSD of 0.8% from five successive measurements using the same Cu-Cy/CTS/GC electrode (Figure S3B). To assess the long-term stability of the Cu-Cy/CTS/GCE, we used a sensor stored at 4 °C in a refrigerator to measure glucose concentrations ranging from 0.1 mM to 0.9 mM every five days (Figure 5D). Over a period of time, the amperomeric current showed only trivial fluctuations, and the Cu-Cy/CTS/GCE still maintained 92.1% of its original current response after 15 days.

### 3.4. Analysis of Real-Life Samples

To further assess the sensor’s practicality in detecting glucose in real samples, we employed the designed Cu-Cy/CTS/GCE to measure glucose levels in serum. Fifty µL of serum samples was added to the 30 mL 0.1 M NaOH solution, then 10 μL 0.1 M glucose was added to the solution. The spiked recoveries were calculated from the standard curve to be 95.7% (Table S1). These findings sufficiently attest to the feasibility of utilizing the CuCy/CTS/GCE-based non-enzymatic glucose sensor.

### 3.5. Portable Direct Detection of Real Samples

**Figure 6:**
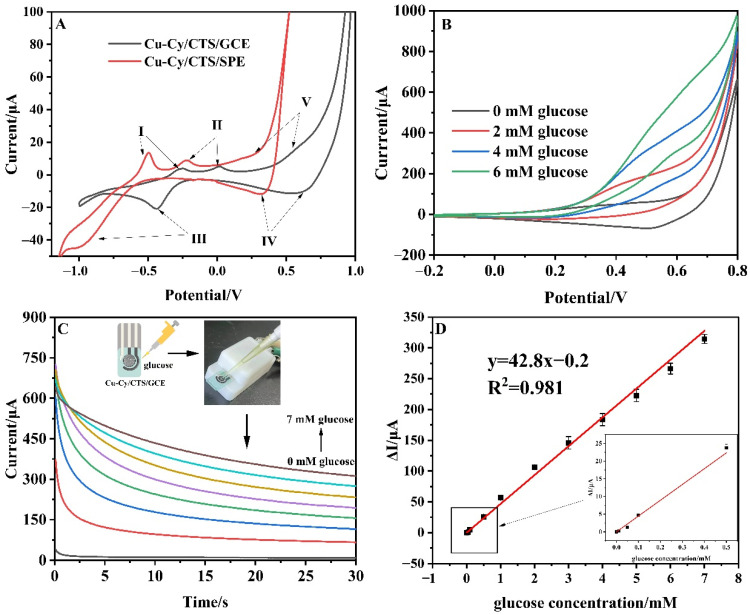
(**A**) CVs of Cu-Cy/CTS/SPE and Cu-Cy/CTS/GCE in 0.1 M NaOH (scan rate: 50 mV/s, I–V: the position of different redox peaks on the electrode surface); (**B**) CVs of Cu-Cy/CTS/SPE containing different concentrations of glucose in 0.1 M NaOH (scan rate: 50 mV/s); (**C**) Amperometric response of Cu-Cy/CTS/SPE electrode upon the addition of various concentrations of glucose at 0.45 V in the range of 1 mM–7 mM; (**D**) The calibration curve between the response current and glucose concentration.

A fabrication method for a non-enzymatic glucose sensor based on screen-printed electrodes has the advantages of simple structure, convenient operation, low cost, and high detection sensitivity [41]. On the basis of the above research, the Cu-Cy/CTS/SPE was further developed for portable direct detection of glucose in the real samples. SPE consists of a carbon working electrode (diameter 4 mm), a carbon counter electrode, and a pseudoreference. The electrode required no pre-treatment, and its modification process was consistent with that of the GCE. As shown in Figure 6A, the redox peaks of Cu-Cy/CTS on SPE shifted the position of all redox peaks in the negative direction by about 0.2 V compared to the redox peaks on GCE, which was due to the excellent electrical conductivity and electrochemical property of the SPE [51]. The CVs of the Cu-Cy/CTS/SPE with and without glucose are displayed in Figure 6B. The oxidation peak of the modified electrode at around 0.45 V increased significantly after the addition of 2/4/6 mM glucose. As depicted in Figure 6C, to further investigate the electrochemical performance of the Cu-Cy/CTS/SPE for glucose detection, a range of glucose concentrations were detected under the detection potential of 0.45 V by constant potential amperometry. The calibration curve of the amount of current change and glucose concentration is displayed in Figure 6D. The Cu-Cy/CTS/SPE exhibited a high sensitivity of 340.76 μA mM^−1^cm^−2^ in the range of 0.01 mM–7 mM (R^2^ = 0.981), which allowed for the direct detection of human serum (3.9 mM–7 mM) and body fluids (50 μM–200 μM). The results in Figure S4A–D show that the Cu-Cy/CTS/SPE also had good reproducibility, with an RSD of 1.5% for five modified electrodes, good repeatability, with an RSD of 4.2% for five successive measurements, long-term stability (maintaining 90.1% of its original current response after 15 days), as well as immunity to interference. The performance of the Cu-Cy/CTS/SPE in blood serum samples was tested. Serum samples did not require dilution; the amount of glucose in the blood can be directly estimated using the calibration curve. According to the literature [33,34], the standard addition method was used in the following measurements. Fifty μL of 0.1 M NaOH and 100 μL of serum samples were sequentially dropped onto the carbon working electrode surface of the SPE. Subsequently, 100 μL of 2 mM glucose was added to the surface of the SPE, and the spiked recoveries were calculated from the standard curve. The recovery values were calculated to be 102.7%, with a relative standard deviation (RSD) of 3.12% (Table S2).

## 4. Conclusions

This study investigated the electrochemical activity of Cu-Cy/CTS/GCE and its electrocatalytic action on glucose through electrochemical methods. A novel electrochemical sensor, based on Cu-Cy, was developed, and its assorted properties were scrutinized. The Cu-Cy/CTS/GCE exhibits outstanding performance in electrochemically oxidizing glucose, featuring a highly sensitive current response. The device demonstrates high accuracy and commendable long-term stability. The Cu-Cy/CTS/GCE showcases remarkable selectivity for the specific detection of glucose amidst significant interferents. Based on these results, a Cu-Cy/CTS/SPE was further developed for portable direct detection of glucose in the real samples. These characteristics highlight a promising future for the Cu-Cy/CTS-modified electrodes in the development of an enzyme-free glucose sensor. On the other hand, the activation of the electrode plays a very important role in catalytic glucose oxidation. In this study, the current activation time was long (about 30 min), so there is room for improvement.

## Figures and Tables

**Table 1:** Comparison of important parameters of the enzyme-free glucose sensor based on Cu-Cy with different glucose sensors based on Cu.

Electrode Materials	Sensitivity (μA·mM^−1^·cm^−2^)	Linear Range (mM)	LOD (μM)	Ref.
Cu nanoparticles modified as-grown CVD graphene	379.31	0.02–2.3	1.39	[44]
Cu-MOF/MWCNTs/GCE	3.878	0.50–11.84	0.40	[45]
Cu-Cu_2_S/GCE	5.02	0.10–500	0.33	[46]
Cu_2_O biscuit/SPCE	309	0.0005–4.03	0.1	[47]
Cu_2_O/AlOOH/rGO	155.1	0.005–14.77	2.6	[48]
Cu-Co/rGO	240	0.001–4	0.15	[49]
Cu-Cy/CTS/GCE	588.28/124.42	0.0027–1.3/ 1.3–7.7	0.28	This work

## Data Availability

Data are contained within the article.

## Supplementary Materials

The following supporting information can be downloaded at: https://www.mdpi.com/article/10.3390/nano14171430/s1, Figure S1: (A) CVs of the Cu-Cy/CTS/GCE at different scan rates from 100 to 1000 mV/s in absence of glucose. Inset shows the relationship of the oxidation peak current with the scan rate, (B) CVs of the Cu-Cy/CTS/GCE at different scanning circles in 0.1 M NaOH solution, (C) CVs of the Cu-Cy/CTS/GCE with immobilization of different concentrations of Cu-Cy in 0.1 M NaOH solution at 50 mV/s; Figure S2: The amperometric responses of Cu-Cy/CTS/GCE in successive additions of 0.1333 mM glucose under various conditions: with different scanning cycles (A), at different detection potential (B), with different concentrations of Cu-Cy(C), and with different concentrations of CTS (D); Figure S3: (A) Reproducibility studies of Cu-Cy/CTS/GCE in the determination of glucose (0.9 mM), (B) Repeatability studies of Cu-Cy/CTS/GCE in the determination of glucose (0.9 mM); Figure S4: (A) Reproducibility studies of Cu-Cy/CTS/SPE in the determination of glucose (1 mM), (B) Reproducibility studies of Cu-Cy/CTS/SPE in the determination of glucose (1 mM), (C) Long-term stability studies of Cu-Cy/CTS/SPE in the determination of glucose (1 mM), (D) Amperometric response of Cu-Cy/CTS/SPE for the successive addition of 1 mM of glucose and 0.1 mM of ascorbic acid (AA), uric acid (UA), NaCl, KCl, lactic acid, glucose in 0.1 M NaOH at applied potential of 0.45 V; Table S1: Determination glucose with Cu-Cy/CTS/GCE in real samples; Table S2: Determination glucose with Cu-Cy/CTS/SPE in real samples.
