# Supplementary material for: A Biocompatible, Highly Sensitive, and Non-Enzymatic Glucose Electrochemical Sensor Based on a Copper-Cysteamine (Cu-Cy)/Chitosan-Modified Electrode
Source: Nanomaterials (Basel). 2024 Aug 31;14(17):1430. doi: 10.3390/nano14171430 (PMC11397198; doi:10.3390/nano14171430)
Supplement: Supplementary file 1 [file nanomaterials-14-01430-s001.zip › nanomaterials-3152066-supplementary.pdf]

Supplementary Material

Manuscript entitled "A biocompatible, highly sensitive, and non-enzymatic glucose electrochemical sensor based on a copper-cysteamine (Cu-Cy)/chitosan modified electrode"

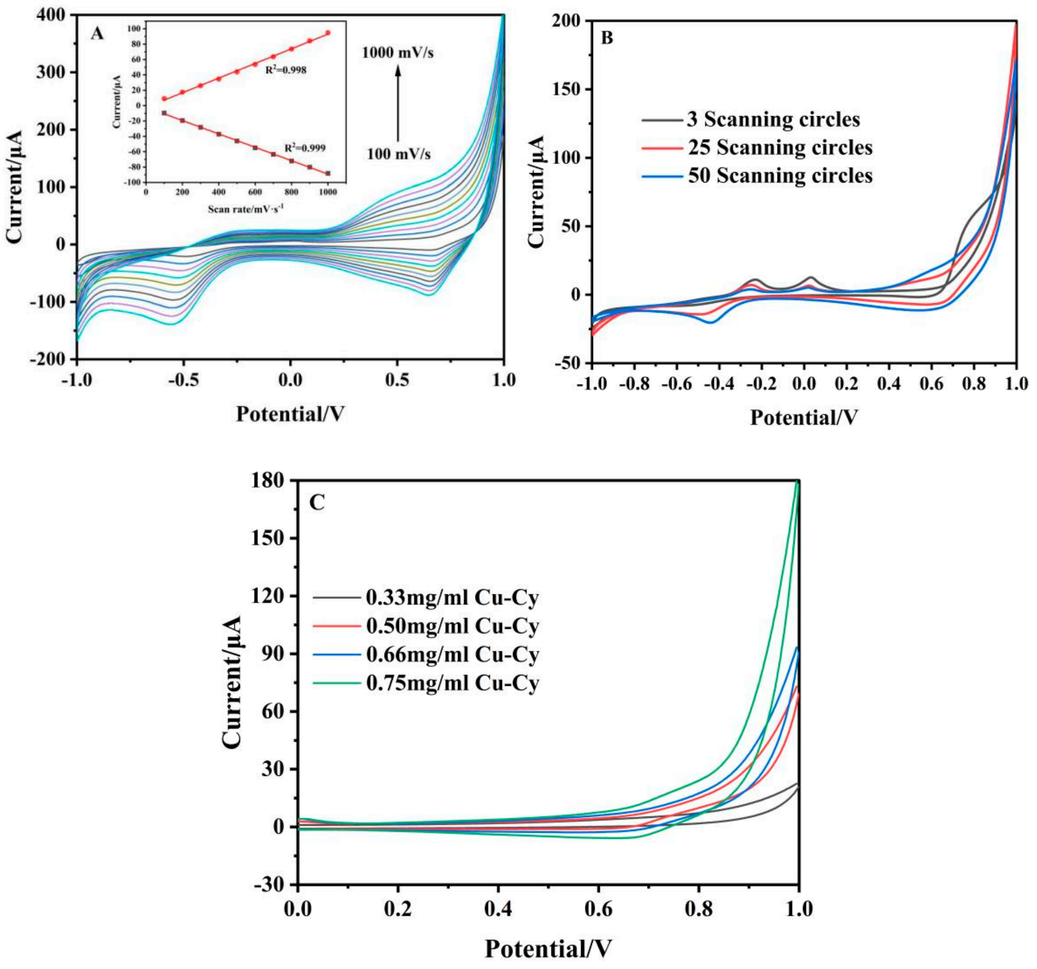

Figure S1. (A) CVs of the Cu-Cy/CTS/GCE at different scan rates from 100 to 1000 mV/s in absence of glucose. Inset shows the relationship of the oxidation peak current with the scan rate, (B) CVs of the Cu-Cy/CTS/GCE at different scanning circles in 0.1 M NaOH solution, (C) CVs of the Cu-Cy/CTS/GCE with immobilization of different concentrations of Cu-Cy in 0.1 M NaOH solution at 50 mV/s.

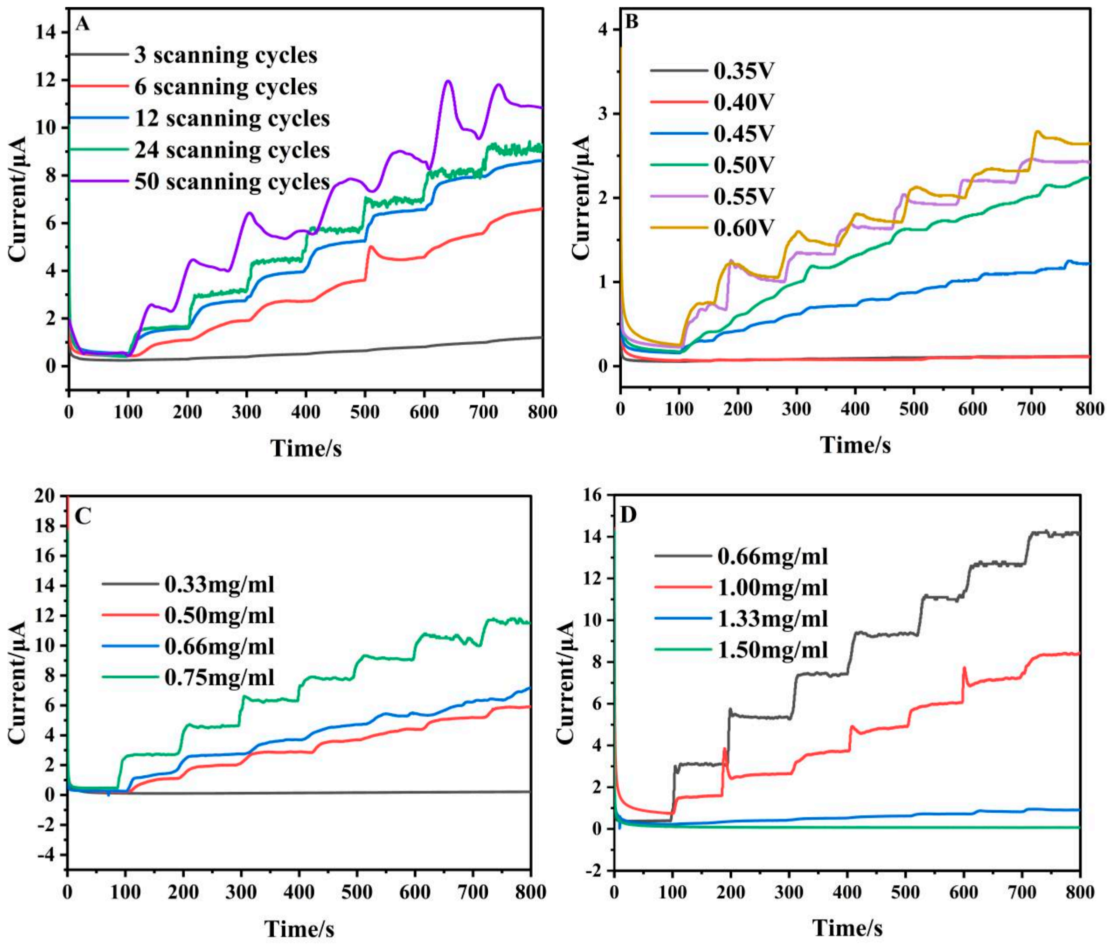

Figure S2. The amperometric responses of Cu-Cy/CTS/GCE in successive additions of 0.1333 mM glucose under various conditions: with different scanning cycles (A) , at different detection potential (B), with different concentrations of Cu-Cy(C), and with different concentrations of CTS (D).

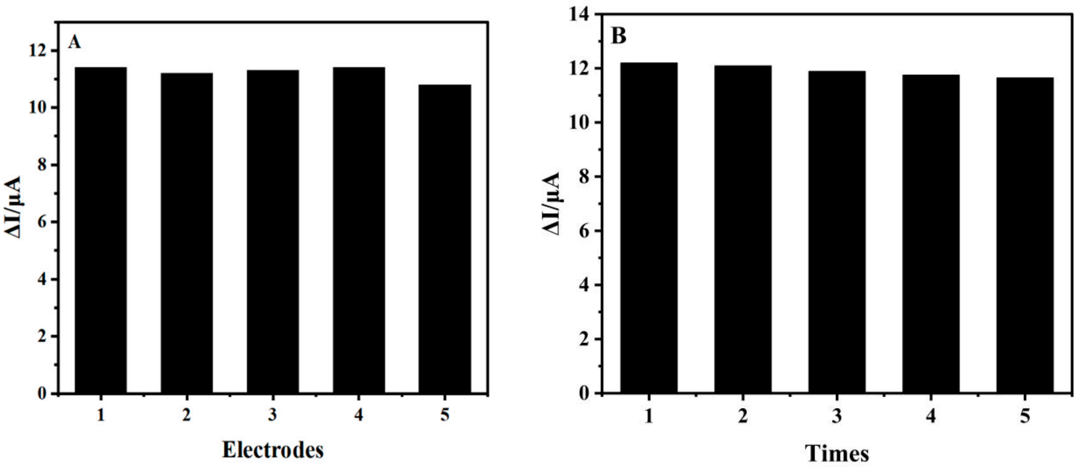

Figure S3. (A) Reproducibility studies of Cu-Cy/CTS/GCE in the determination of glucose (0.9mM), (B)Repeatability studies of Cu-Cy/CTS/GCE in the determination of glucose (0.9mM).

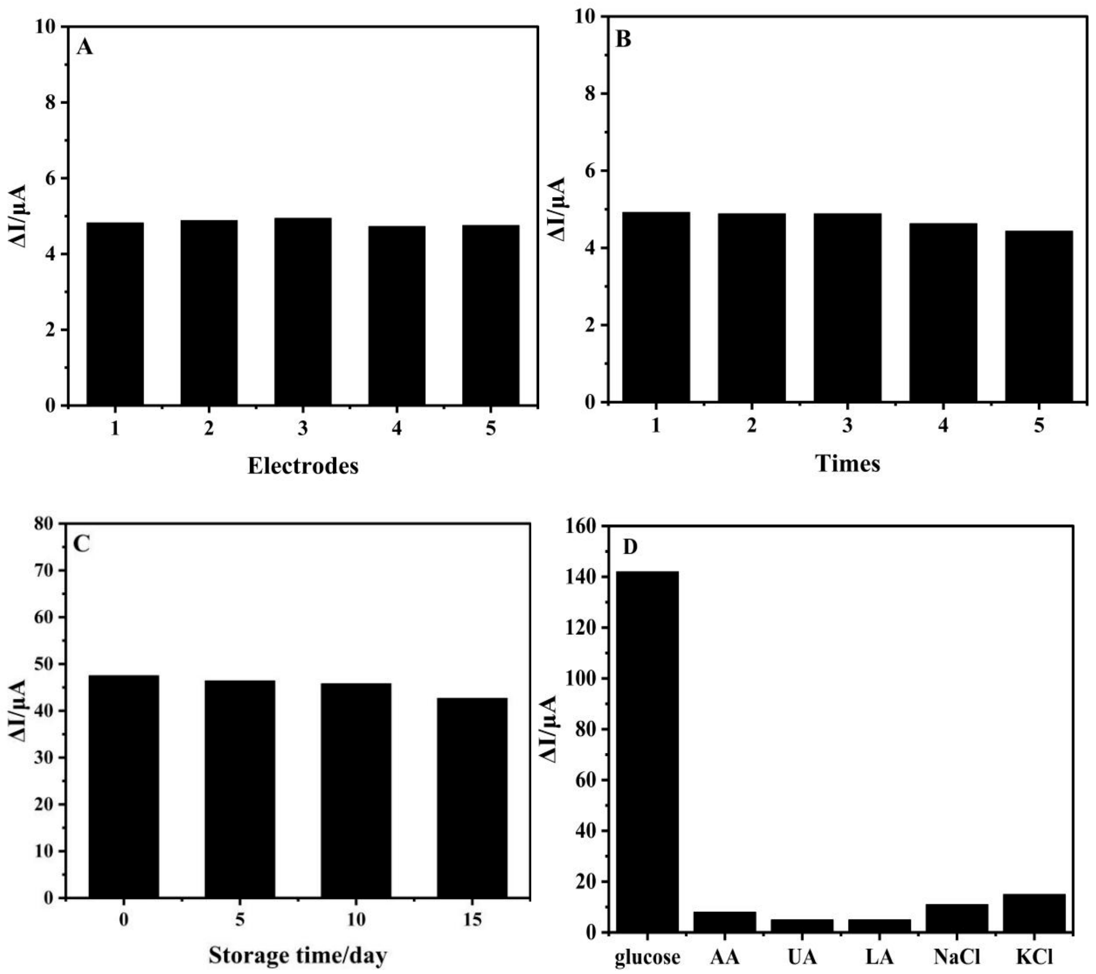

Figure S4. (A) Reproducibility studies of Cu-Cy/CTS/SPE in the determination of glucose (1mM), (B) Reproducibility studies of Cu-Cy/CTS/SPE in the determination of glucose (1mM), (C) Long-term stability studies of Cu-Cy/CTS/SPE in the determination of glucose (1mM), (D)Amperometric response of Cu-Cy/CTS/SPE for the successive addition of 1mM of glucose and 0.1mM of asco rbic acid (AA), uric acid (UA), NaCl, KCl, lactic acid, glucose in 0.1 M NaOH at applied potential of 0.45 V.

Table S1 Determination glucose with Cu-Cy/CTS/GCE in real samples .

Samples	Added (mM)	Founded (mM)	RSD (n=3)	Recovery
1	~	0.0088	2.27	
2	0.0333	0.0423	3.87	95.7%

Table S2 Determination glucose with Cu-Cy/CTS/SPE in real samples .

Samples	Added(mM)	Founded(mM)	RSD(n=3)	Recovery
1	~	3.29	4.79	
2	2.0	5.25	3.12	102.7%
